# Hybrid Photoelectrocatalytic TiO_2_-Co_3_O_4_/Co(OH)_2_ Materials Prepared from Bio-Based Surfactants for Water Splitting

**DOI:** 10.3390/molecules28227599

**Published:** 2023-11-15

**Authors:** Fanny Duquet, Valérie Flaud, Christina Villeneuve-Faure, Matthieu Rivallin, Florence Rouessac, Stéphanie Roualdès

**Affiliations:** 1 Institut Européen des Membranes (IEM), UMR 5635, University of Montpellier, ENSCM, CNRS, 34095 Montpellier, France; florence.rouessac@umontpellier.fr (F.R.); stephanie.roualdes@umontpellier.fr (S.R.); 2 Institut Charles Gerhardt Montpellier (ICGM), UMR 5253, University of Montpellier, ENSCM, CNRS, 34095 Montpellier, France; valerie.flaud@umontpellier.fr; 3 LAPLACE (Laboratoire Plasma et Conversion d’Energie), Université de Toulouse, UPS, CNRS, INPT, 31062 Toulouse, France; christina.villeneuve@laplace.univ-tlse.fr

**Keywords:** composite materials, photoanodes, water splitting, heterojunctions, titanium oxides, cobalt oxides

## Abstract

The development of new photoanode materials for hydrogen production and water treatment is in full progress. In this context, hybrid TiO_2_-Co_3_O_4_/Co(OH)_2_ photoanodes prepared using the sol–gel method using biosurfactants are currently being developed by our group. The combination of TiO_2_ with a cobalt-based compound significantly enhances the visible absorption and electrochemical performance of thin films, which is mainly due to an increase in the specific surface area and a decrease in the charge transfer resistance on the surface of the thin films. The formation of these composites allows for a 30-fold increase in the current density when compared to cobalt-free materials, with the best TiO_2_-CoN0.5 sample achieving a current of 1.570 mA.cm^−2^ and a theoretical H_2_ production rate of 0.3 µmol.min^−1^.cm^−2^ under xenon illumination.

## 1. Introduction

One of the most attractive solutions for reducing air pollution and slowing down global warming is the use of “green” hydrogen as an energy carrier. By using a photoelectrochemical cell (PEC) at the interface between water electrolysis and photocatalysis, hydrogen can be produced without greenhouse gas emissions [1,2,3]. Moreover, the use of partially polluted water as a resource for this cell would allow for the coupling of green hydrogen production with tertiary water treatment [4,5,6,7,8]. In this respect, work on the components of this cell, especially the photoanodes, is essential to make such a system competitive.

The photoanode plays a key role in a PEC. It receives the light and generates the charges necessary for the oxidation reaction of the water on its surface and, consequently, for the production of hydrogen at the counter electrode, as well as for the mineralization of the organic pollutants present in the water (in the case of polluted water as a resource). It must be of the semiconductor type, and our choice fell on TiO_2_, which is already known for its photocatalytic properties, among its other advantages [9,10,11]. However, TiO_2_ has disadvantages, such as low absorption in the solar spectrum (limited to the UV range), rapid charge recombination, and low electronic conductivity [12,13,14]. The formation of a heterojunction combining the n-type TiO_2_ semiconductor with a p-type semiconductor can improve these properties [15,16,17,18]. Cobalt oxide has already proven itself as a dopant for TiO_2_ due to its high catalytic activity, low cost, stability in neutral and alkaline environments, and ability to be used as a bifunctional electrode [19,20]. Co_3_O_4_ has interesting electronic properties, which are advantageous for our application, and also a direct band gap between 1.5 and 2.5 eV, which is essential for improving absorption in the visible range [21,22,23,24,25]. Thus, this p-type semiconductor was chosen in this study to realize an n-TiO_2_│p-Co_3_O_4_-type heterojunction.

This kind of heterojunction has demonstrated enhanced electrochemical performance through diverse synthesis routes, such as the hydrothermal route [26,27], anodization [28], atomic layer deposition [29], metal organic chemical vapor deposition [30], and impregnation [31]. Regarding the sol–gel synthesis, it is rarely the only route used for n│p materials, but it is often coupled with other synthesis routes. In this study, our goal was to create photoanodic materials using titanium and cobalt oxides along with different bio-sourced surfactants via a new one-step sol–gel method. This synthesis method is cost-effective, easy to implement, and could be transferred to other types of heterojunctions, allowing for a broader study. We decided to use three different surfactants and to introduce cobalt oxide in two molar ratios (HNO_3_/TTIP/Co(NO_3_)_2_•6 H_2_O = 1/1/0.25 and 1/1/0.5), to allow for a high degree of modularity in terms of the chemical composition, structural properties and, therefore, the functional properties of the photoanodes, in order to optimize their performance in water splitting.

## 2. Results and Discussion

### 2.1. Structural and Textural Characterizations

**Figure 1:**
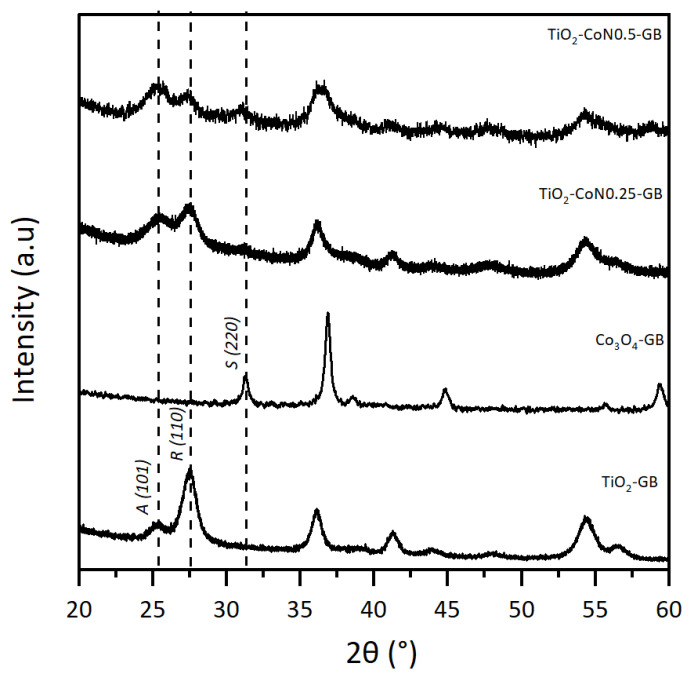
Powder diffraction patterns for TiO_2_-GB, CO_3_O_4_-GB, TiO_2_-CoN0.25-GB, and TiO_2_-CoN0.5-GB.

The TiO_2_/Co_3_O_4_ samples prepared with the GB surfactant were selected to illustrate the XRD powder results, shown in Figure 1, with the diffractograms of TiO_2_-GB, TiO_2_-CoN0.25-GB, TiO_2_-CoN0.5-GB, and CO_3_O_4_-GB. In order to compare the crystal structures of the TiO_2_-Co_3_O_4_ hybrid powders to those of the individual oxides, a Co_3_O_4_ powder (1:1:1 ratio) was synthesized using the same procedure as described above. On the TiO_2_-GB diffractogram, the presence of TiO_2_ characteristic peaks was noted at 25.4° for the anatase phase (A), and at 27.5° for the rutile phase (R) [32]. For the Co_3_O_4_-GB powder, the characteristic peak of the spinel phase (S) was observed at 31.3° [33].

For the XRD analysis, we used a copper (Cu) anticathode. The presence of cobalt (Co) in our samples, an element close to Cu in the periodic table, leads to a fluorescence phenomenon due to an absorption threshold close in energy to that of the incident beam. The resulting diagram exhibits a high continuous background with high noise, which obstructs the proper observation of the Co_3_O_4_ peaks in the composite materials diffractograms (TiO_2_-CoN0.25-GB and TiO_2_-CoN0.5-GB). However, the three phases of the hybrid powder are observable, and it can be seen that the addition of Co_3_O_4_ does not affect the position of the diffracted TiO_2_ peaks, but does appear to affect the anatase/rutile phase ratio.

**Figure 2:**
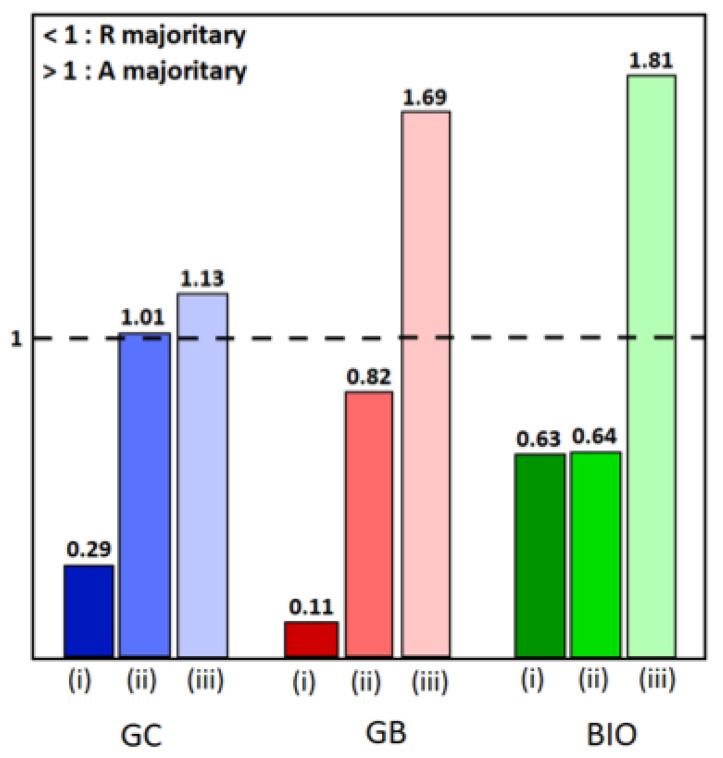
Histograms of anatase/rutile ratios: (**i**) for TiO_2_-type samples, (**ii**) for TiO_2_-CoN0.25-type samples, and (**iii**) for TiO_2_-CoN0.5-type samples, in blue, red and green colors for GC, GB and BIO surfactants, respectively.

To know more, the anatase and rutile proportions were calculated using the Rietveld refinement, and are reported in the Supplementary Materials Table S1 [34]. As already displayed in our previous work, the rutile phase predominates in the TiO_2_ powders, but the anatase/rutile ratio varies depending on the surfactant used [35]. For the hybrid powders, the influence of the addition of cobalt oxide on the TiO_2_ phases is illustrated in the histogram (Figure 2). It can be seen that the presence of the spinel phase causes a decrease in the proportion of the rutile phase in favor of the anatase phase, to the point where it becomes dominant. It appears that the presence of Co_3_O_4_ inhibits the effect of the surfactant on the formation and stability of the anatase phase [36,37,38].

The calculation of the crystallite size was performed using the Scherrer Formula (1), with *K* as the form factor (0.9 value for a spherical crystallite shape), *λ* as the CuKα wavelength (in nanometers), HW as the half-width value (in radians), and *θ* as the corresponding diffraction angle (in radians). The results suggest that cobalt oxide has a direct impact on the formation and size of the anatase and rutile crystallites (refer to Table S2).

The crystallite size of the hybrid powders decreases independently of the type of surfactant, reaching values centered on 4 nm for the anatase phase and 6 nm for the rutile phase. The amount of cobalt oxide does not seem to have any influence, since the two ratios, 0.25 and 0.5, induce similar crystallite size values. It can be concluded that the formation of the hybrid material containing the TiO_2_/Co_3_O_4_ phase mixture allows for a smoothing of the crystallite size of the different TiO_2_ phases.

(1) $$d=\frac{K\times \lambda }{HW\times \cos\theta }=\frac{0.9\times 0.15406}{HW\times \cos\theta }$$

The BET isotherms for the hybrid materials show the same pattern as observed for TiO_2_-GC in the previous work (Figure S1), indicating the dual presence of microporosity and mesoporosity. Table 1 shows the specific surface areas, average sizes, and pore distributions of the materials. The specific surface area of the materials formed by the two oxides is higher than that of the TiO_2_ powders, and increases with the amount of cobalt oxide. In fact, we found the following hierarchy, TiO_2_-CoN0.5 > TiO_2_-CoN0.25 > TiO_2_, in terms of the specific surface area. This increase is particularly remarkable for the GB and BIO samples, which reached specific surface areas of 97 and 126 m^2^.g^−1^, respectively, for the highest Ti/Co ratio (0.5). For the GC, whose specific surface area without cobalt was by far the highest, the increase was less, but the threshold of 126 m^2^.g^−1^ was reached for the same ratio (0.5). However, the average pore size of the hybrid materials did not change much between the different samples, making it difficult to correlate with the specific surface area. It seems that, as with the crystallite size above, the pore size for all the TiO_2_-Co_3_O_4_ materials homogenized with the addition of cobalt, to an average value of about 4.7 nm. The difference in the specific surface area despite the almost identical pore size could be explained by a difference in the size of the “mixed” aggregates.

The pore size distribution for the TiO_2_-CoN0.25 materials is similar, with all three samples showing a bimodal distribution centered at around 3.5 nm and 5 nm. A monodispersity is observed for the 3.5 nm pore size, and a polydispersity for the 5 nm pore size (Figure S2). The same observation can be made for the TiO_2_-CoN0.5-type materials, which have the highest cobalt loading, with the three samples showing the same type of distribution. For the 0.5 ratio, a unimodal and monodispersed distribution centered at around 3.5 nm for the GC and GB, and closer to 3 nm for the BIO (Figure S3), can be observed. Through the pore size distribution, we again find a homogenization effect on the properties of the samples due to the addition of cobalt oxide. Based on the above results, only the samples synthesized with the GC surfactant with the highest specific surface area (TiO_2_-GC, TiO_2_-CoN0.25-GC, and TiO_2_-CoN0.5-GC) are further characterized in the following.

### 2.2. Morphological and Chemical Characterizations

**Figure 3:**
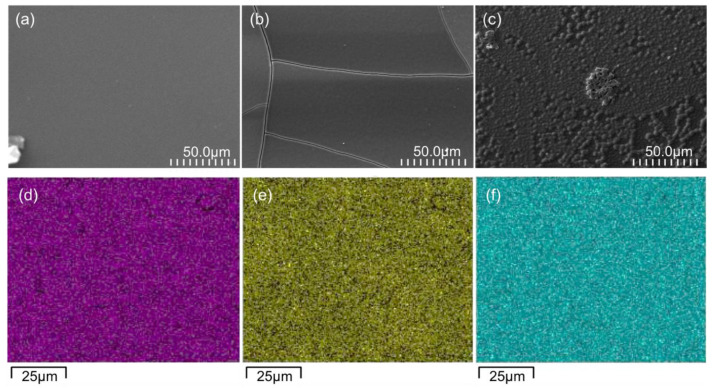
SEM images of the surfaces of (**a**) TiO_2_-GC, (**b**) TiO_2_-CoN0.25-GC, and (**c**) TiO_2_-CoN0.5-GC. EDX mapping of TiO_2_-CoN0.5-GC sample: in pink (**d**), distribution of Ti element; in yellow (**e**), Co element; and, in blue (**f**), O element.

The SEM images of the surfaces of the three GC-based thin films deposited on the ITO support are shown in Figure 3. The surface of the TiO_2_-GC thin film (Figure 3a) is smooth and free of imperfections, which is not the case after the addition of Co_3_O_4_. At a ratio of 0.25 (Figure 3b), cracks appear, probably due to a change in the surface tension caused by the addition of Co, but the surface of the thin film remains smooth.

On the other hand, when the ratio is increased to 0.5 (Figure 3c), the appearance of the thin film surface is completely changed. A surface consisting of aggregates of TiO_2_ and Co_3_O_4_ nanoparticles is observed. This was confirmed with a 5 µm × 5 µm surface topography measurement using AFM, which demonstrated an increase in the arithmetic surface roughness from 0.8 nm for the TiO_2_, to 3.8 nm and 11.5 nm for the Co_3_O_4_ ratios of 0.25 and 0.5, respectively. The thicknesses of the thin films are approximately the same, with a total value of 1100 ± 350 nm.

The EDX analysis indicates that the Ti, Co, and O elements are uniformly distributed throughout the TiO_2_-CoN0.5-GC thin film (Figure 3d–f). This observation is also true for the other TiO_2_-CoN0.25-GC thin film. Via a semi-quantitative study of the EDX maps, the elemental composition ratios of Co/Ti for the TiO_2_-CoN0.25-GC and TiO_2_-CoN0.5-GC samples were determined to be 0.27 and 0.59, respectively.

**Figure 4:**
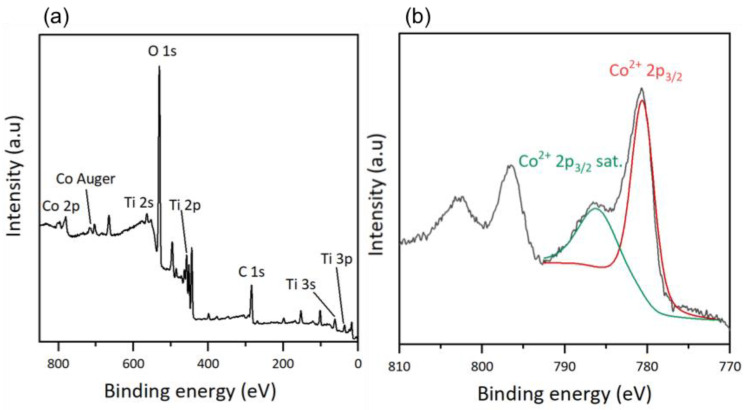
XPS results: (**a**) survey of TiO_2_-CoN0.5-GC and (**b**) Co2p scan after deconvolution of TiO_2_-CoN0.5-GC.

An XPS analysis was performed to determine the surface composition of the thin films. For the hybrid materials, the total spectrum of the TiO_2_-CoN0.5-GC sample is shown in Figure 4a, and is similar to those of the other samples, regardless of the ratio used. For the total spectrum, we observed the presence of the same chemical elements as in the TiO_2_ thin films (C, O, Ti and Sn, and In from the ITO substrate) and, in addition, the cobalt Co 2p appears at high binding energies [35]. The Co Auger peak corresponds to the emission of an electron during the de-excitation of the Co atom.

To verify the nature of the cobalt on the surface of the thin films, a high-resolution deconvolution of the Co 2p spectrum was performed (Figure 4b). The main peak related to Co^2+^ 2p_3/2_ at 780.5 eV and its satellite peak at 786.0 eV could be identified. The presence of a satellite peak is characteristic of Co^2+^, with an outgoing electron interacting with a valence electron and exciting it, thereby shaking it to a higher energy level. As a consequence, the energy of the core electron is reduced and a satellite structure appears a few eV below the core level position; it is a shake-up-type peak satellite [39,40]. Cobalt oxide Co_3_O_4_ is a mixed-valence compound, and the cobalt exists in two oxidation states: Co^2+^ and Co^3+^. However, the deconvolution shows only the presence of Co^2+^, suggesting that cobalt in its oxidation state (III) does not exist on the surface of the mixed thin films. According to Biesinger, M. C. et al., the position and shape of the Co^2+^ 2p_3/2_ peak indicates the presence of cobalt in the form of Co(OH)_2_ [41]. Since the XPS gives access to a surface measurement, the cobalt oxide had probably adsorbed water molecules, inducing the formation of Co-O bonds, with the oxygen coming from the water molecules. This hydroxide form on the surface of the materials applies to both mixed materials, regardless of the Co ratio.

**Figure 5:**
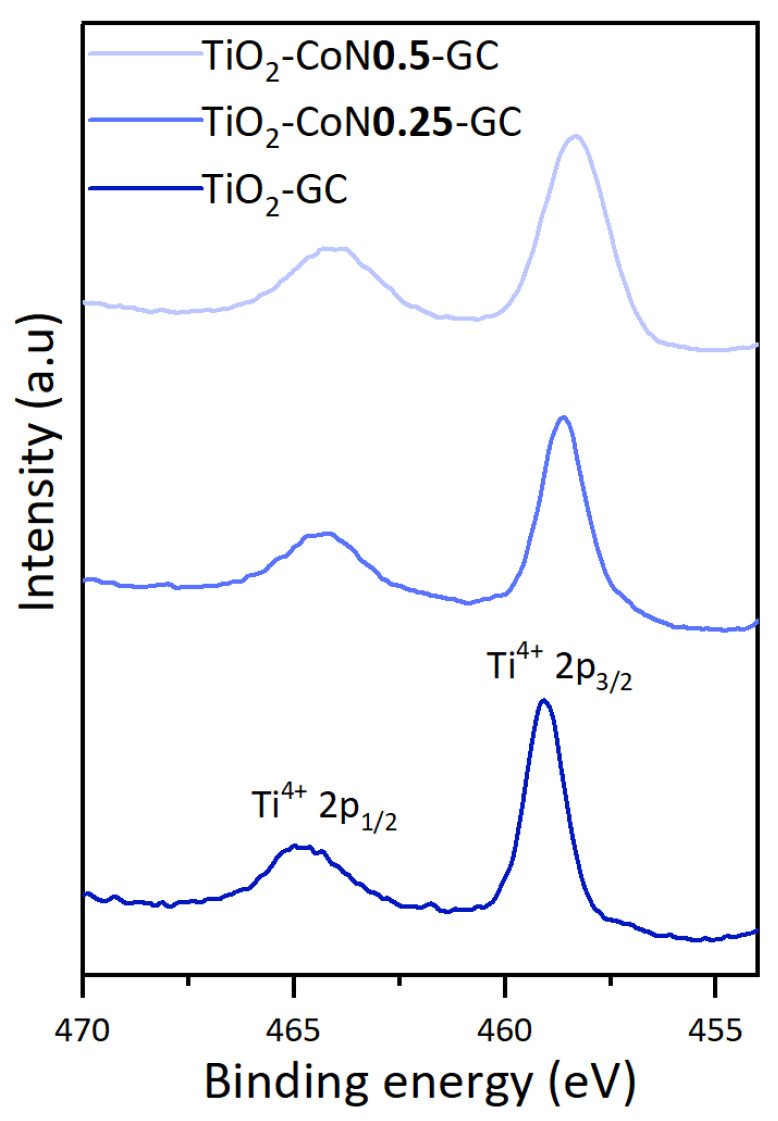
Comparison of Ti 2p scan of the three thin films.

To evaluate the effect of the addition of cobalt oxide on the titanium oxide, the Ti 2p spectra for the three samples synthesized from the GC surfactant are shown in Figure 5.

The Ti 2p spectra display two symmetrical peaks corresponding to Ti 2p_1/2_ and Ti 2p_3/2_, representing Ti^4+^ in TiO_2_. With the incorporation of Co, there is a subsequent shift to lower binding energies (+0.5 eV for the TiO_2_-CoN0.25 and 0.2 eV for the TiO_2_-CoN0.5), indicating the occurrence of an electron transfer between the Co_3_O_4_/Co(OH)_2_ and TiO_2_. This transfer establishes an interaction between them, which can facilitate surface charge separation and enhance the photocatalytic activity of the thin films [42,43].

### 2.3. Optical Properties

**Figure 6:**
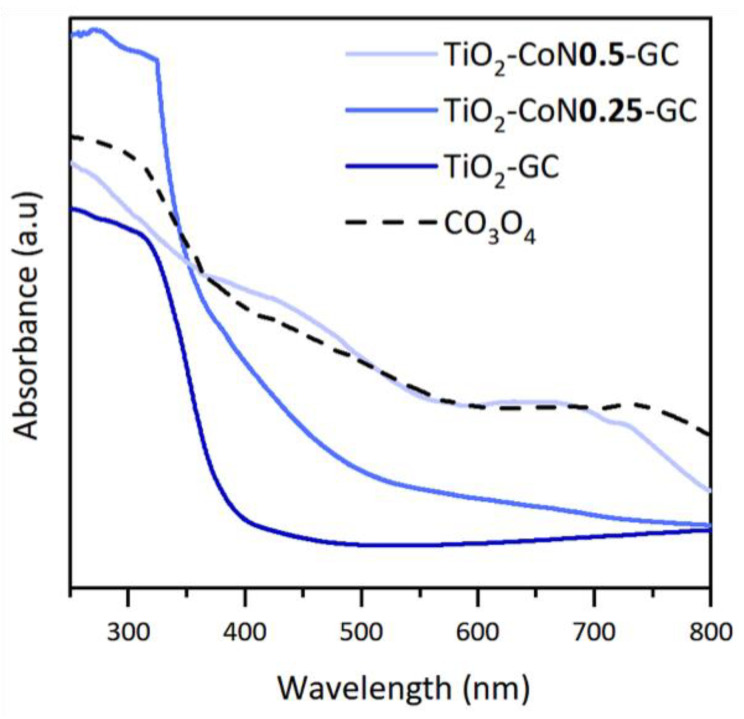
UV-visible spectroscopy data with the absorption spectra of the GC samples and Co_3_O_4_.

In order to determine the evolution of the absorption of the thin films in the visible region, The UV-visible-NIR spectra were studied. TiO_2_ is an oxide that absorbs mainly in the UV region (from 100 to 400 nm), while Co_3_O_4_ absorbs in the visible region (from 400 to 780 nm). Figure 6 shows the absorption spectra of the samples. The TiO_2_ thin film spectrum is characteristic of TiO_2_, with absorption at wavelengths below 400 nm. With the addition of a small amount of cobalt oxide, a shift in the absorption towards the visible region is observed, as well as an increase in absorbance compared to the cobalt-free sample.

The absorption spectrum of the TiO_2_-CoN0.5 thin film shows that increasing the Co content reinforces the above observations. Therefore, it can be concluded from the absorption spectra that the addition of cobalt oxide has the desired effect of increasing the absorption in the visible region for the TiO_2_-CoN0.25 and TiO_2_-CoN0.5 samples. Figure 6 shows that the absorption profile of the cobalt oxide is similar to that of the previously highlighted TiO_2_-CoN0.5 thin films, indicating that the cobalt oxide is at the origin of the improvement in the optical properties of the mixed materials. To determine the band gap of the thin films, a Tauc diagram was constructed by plotting the coefficient ($\alpha hv{)}^{n}$ as a function of energy, E, in eV. The value of the band gap was obtained by extrapolating the linear portion of the curve at the intersection of this line with the x-axis.

The absorption coefficient α (cm^−1^) and the frequency ν (Hz) can be determined from Equations (2) and (3), where A is the absorbance, e is the thickness of the thin film (cm), c is the speed of light (m.s^−1^), and λ is the wavelength (m).

(2) $$\alpha =\frac{A}{100}\times \frac{1}{e}$$

(3) $$v=\frac{c}{\lambda }$$

The coefficient *n* is related to the type of band gap; namely, *n* = 0.5 for an indirect band gap (anatase phase) or *n* = 2 for a direct band gap (rutile and spinel phases).

The energy E (eV) can be determined using Equation (4), where q is the elementary charge (1.6 × 10^−19^ C) and h is Planck’s constant (6.63 × 10^−34^ J.s):

(4) $$E=\frac{h\nu }{q}=\frac{hc}{\lambda q}$$

The theoretical value of the indirect band gap of the anatase phase is 3.2 eV, and that of the direct band gap of the rutile phase is 3.0 eV. For the spinel phase of cobalt oxide, the direct band gap is between 1.5 and 2.5 eV. It is interesting to compare these theoretical values to the experimental values determined using the Tauc plot, distinguishing between the indirect and direct band gaps. For the TiO_2_ thin film, an indirect band gap lowers the value of the anatase phase obtained, with a value of 2.87 eV. This difference between the experimental and theoretical values is not surprising, considering the small amount of anatase crystal phase in the compound (22%). For the direct band gaps, the experimental value is 3.37 eV, which is higher than the theoretical value for the rutile phase. For the TiO_2_-CoN0.25 thin film, the indirect band gap shows values close to those of the indirect band gap of the TiO_2_ film, with a slight decrease. For the direct band gap, two energy levels can be displayed. The first energy level (2.73 eV) is close to the value of the cobalt oxide band gap, while the second energy level (3.54 eV) is close to the theoretical value for the rutile phase. Therefore, the band gaps of the three crystalline phases present in the TiO_2_-CoN0.25-GC can be effectively observed. Finally, the TiO_2_-CoN0.5-GC film has a band gap value of 3.11 eV, which is close to that of the rutile phase, which is not the dominant phase of this material (A: 37%, R: 33%, and S: 30%).

It is, therefore, difficult to attribute this value to either the rutile or spinel phase. However, given the previous values found for the rutile phase (3.37 eV and 3.54 eV) and for the spinel phase (2.73 eV), we can imagine that the value of 3.11 eV is an intermediate value that is effectively representative of the band gap of this material. In this type of thin film (with a high cobalt content), no reliable values for the indirect band gap of the anatase phase could be obtained. Finally, the calculations of the band gap values from the Tauc diagrams confirmed the presence of the different crystalline phases and the effect of cobalt oxide: the addition of cobalt oxide to the materials lowered the band gap energy and, thus, shifted the absorption of the materials into the visible region.

### 2.4. Electronic Properties

In order to compare the electronic conductivity of thin films at the nanometer scale, current measurements were made at negative voltages using C-AFM. For an n-type semiconductor such as TiO_2_, the current flows best at negative voltages; conversely, for a p-type semiconductor such as Co_3_O_4_, the current flows best at positive voltages. However, the position of the energy levels of the band structure of the semiconductor, in relation to the Fermi level of the metal forming the tip (here PtSi), also has an effect on the current collected. The choice of a negative or positive voltage does not necessarily define the nature of the majority carriers, n or p. In our case, it is more relevant to present the results for a negative voltage, since the films mostly contain TiO_2_.

**Figure 7:**
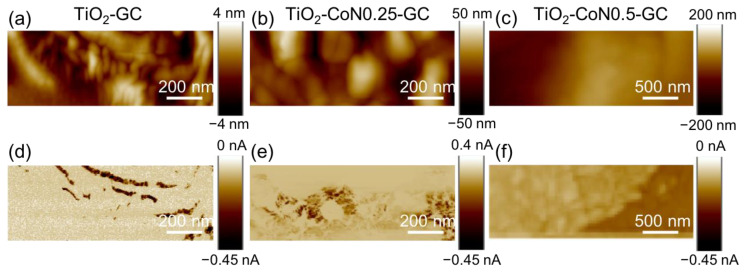
(**a**–**c**) Topography and (**d**–**f**) current maps for the corresponding areas for TiO_2_-GC samples at −5 V, TiO_2_-CoN0.25-GC at −9 V, and TiO_2_-CoN0.5-GC at −9 V, respectively.

In the topography maps shown in Figure 7a–c, the presence of cobalt oxide seems to induce a smoothing of the thin films, with a decrease in crystallite aggregation.

Figure 7d–f represent the corresponding current; the distribution is heterogeneous for all the samples, as can be seen from the current maps with two different colors. The percentage of conductive area for the hybrid thin films increases from 20% for the TiO_2_-GC to 85% and 98% for the TiO_2_-CoN0.25-GC and TiO_2_-CoN0.5-GC, respectively (Table S3).

In contrast to the percentage of conductive area, the collected current in the conductive area (i.e., dark area) decreases with the Co ratio, ranging from −0.45 nA for the TiO_2_-GC to −0.035 nA for the TiO_2_-CoN0.25 and to −0.045 nA/−0.122 nA for TiO_2_-CoN0.5-GC. As can be seen, for the thin film with the highest cobalt ratio, two current values were collected, proving the inhomogeneous current distribution in the bulk. This could be evidence of the presence of a large aggregate of TiO_2_ and Co_3_O_4_ nanoparticles, with the most conductive areas associated with cobalt oxide, which is known to have better electrical conductivity than titanium oxide. Since the conductive surface and the current collected in the conductive surface exhibited different behaviors with the Co ratio, it is important to compare the average current over similar surfaces. This mean current ranged from −71 pA for the TiO_2_ to −28 pA and −47 pA for the 0.25 and 0.5 Co ratios, respectively. Thus, the presence of cobalt oxide did not have the expected effect on the electronic conductivities from a nanometric point of view. In fact, the Co_3_O_4_ had a higher intrinsic conductivity than the TiO_2_. However, this effect was limited by other parameters, such as: (i) the reduction in the crystallite sizes and (ii) the modification of the ratio between the anatase and rutile phases, or the modification of the band gap.

To investigate the electronic conductivity at the interface between the thin film and electrolyte, we employed an electrical model corresponding of our system. This model includes one resistor, Rs, connected in series with two resistor–constant phase element pairs, Rp-1/CPE-p-1 and Rp-2/CPE-p-2, connected in parallel [44].

**Figure 8:**
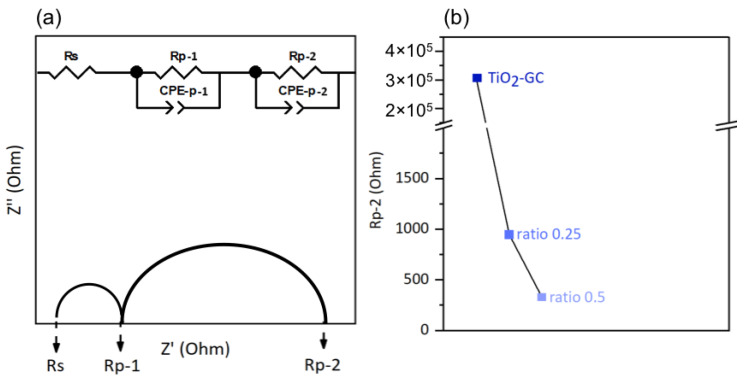
(**a**) Example of a Nyquist plot and illustration of the equivalent circuit model used in this study (Z′ = ReZ and Z″ = −ImZ) and (**b**) charge transfer resistance Rp-2.

The resistor Rs is characteristic of the electrolyte, and the two pairs Rp-1/CPE-p-1 and Rp-2/CPE-p-2 correspond to the ITO support and the supported thin film, respectively. These components are shown in Figure 8a, using a Nyquist plot as an example.

The charge transfer resistances of the electrolyte (Rs) and at the ITO–substrate interface (Rp-1) are not expected to vary from sample to sample because the electrolyte and ITO substrate are the same for all samples.

However, the ITO/thin film interface may slightly affect the Rp-1 value depending on the nature of the thin film in question. The values of Rs and Rp-1 obtained from the Nyquist plots in the dark are listed in Table S4. Variations in these values, depending on the nature of thin film studied, have been observed, but we are unable to explain them. However, we mainly discuss here the charge transfer resistance at the thin film/electrolyte interface (Rp-2), and its evolution according to the nature of the thin films. The lower the Rp-2 resistance, the easier the electron transfer at the material/electrolyte interface, which generally leads to a better electron transfer in the whole electrochemical cell, for a higher faradic efficiency. The onset potential used for the EIS was the minimum potential required to initiate the electrolysis reaction of water. The values of the onset potentials chosen in this study were derived from the cyclic voltammetry curves presented in the following section, and are listed in Table S4. The Nyquist plots of the different samples are shown in Figure S4; the decrease in the circular arc with the addition of cobalt oxide is obvious. The Rp-2 resistance value is shown in Figure 8b for better visualization. For the TiO_2_-GC, the Rp-2 value is 306 771 Ω, which is much higher than that of the TiO_2_-CoN0.25 (954 Ω) or the TiO_2_-CoN0.5 (332 Ω). The decrease in Rp-2 is less obvious when the amount of cobalt oxide is increased, but is still visible.

It can be concluded that the presence of cobalt oxide and the increase in specific surface is a great asset with which to reduce the charge transfer resistance of the hybrid thin films.

### 2.5. Photoelectrochemical Properties

**Figure 9:**
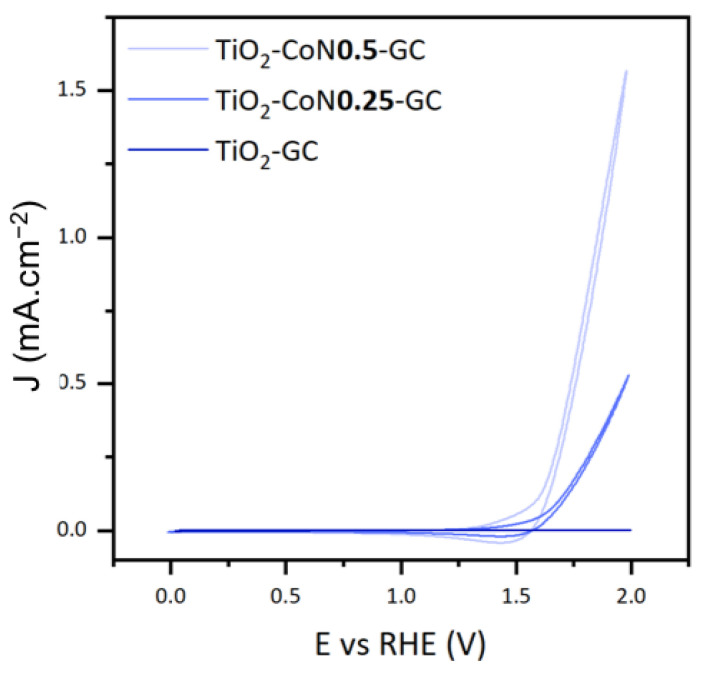
Cyclic voltammetry curves for TiO_2_-GC, TiO_2_-CoN0.25-GC, and TiO_2_-CoN0.5-GC thin films under xenon irradiation.

To study the photoelectrocatalytic performance of the thin films, we focused on three characteristics: the onset potential (E_onset_), the maximum current intensity (I_max_), and the photocatalytic activity of the materials. Figure 9 illustrates the voltammograms collected under xenon irradiation for the three thin films.

The onset potential is defined as the voltage at which the water oxidation reaction initiates, theoretically equivalent to 1.23 V. In practice, this potential can range from 1.8 V to 2.7 V due to cathodic and anodic overvoltages and ohmic losses. The reaction requires less energy as the potential decreases. In this case, the introduction of CO_3_O_4_ decreased the potential significantly, lowering it from 1.84 V in the TiO_2_ thin film to 1.62 V in both the TiO_2_-CoN0.25 and TiO_2_-CoN0.5 composite films.

For the maximum current intensities collected at 2 V, we saw a considerable improvement with the presence of cobalt: as the cobalt ratio increased, the current rose from 0.041 mA.cm^−2^ for the TiO_2_ to 0.529 mA.cm^−2^ for the 0.25 ratio, then to 1.570 mA.cm^−2^ for the 0.5 ratio.

**Figure 10:**
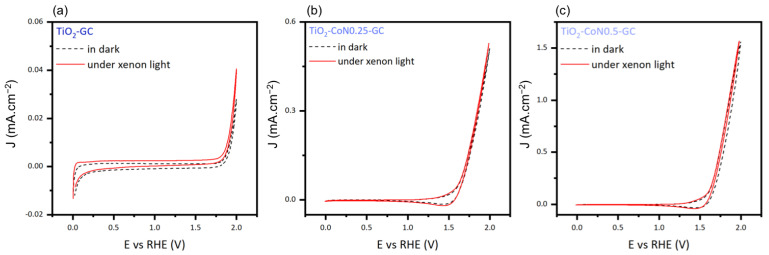
Cyclic voltammetry curves in dark and under xenon light for (**a**) TiO_2_-GC, (**b**) TiO_2_-CoN0.25-GC, and (**c**) TiO_2_-CoN0.5-GC samples.

To determine the photocatalytic activity of the thin films, we measured the difference in current intensity between the dark and xenon irradiation (Figure 10). Comparing the current densities in the dark and under xenon irradiation, the TiO_2_ layer displayed a difference of 0.012 mA.cm^−2^, while the TiO_2_-CoN0.25 and TiO_2_-CoN0.5 exhibited differences of 0.025 and 0.004, respectively. Based on the relative magnitudes of the current intensities, only the TiO_2_ demonstrated significant photocatalytic activity. The presence of the CO_3_O_4_ appears to have interfered with the photocatalytic capabilities of the TiO_2_.

In conclusion, the addition of cobalt and the creation of composite thin films greatly improved the thermodynamic (E_onset_) and kinetic (I_max_) aspects of the water oxidation reaction. This enhancement came at the detriment of TiO_2_ photoactivity.

**Figure 11:**
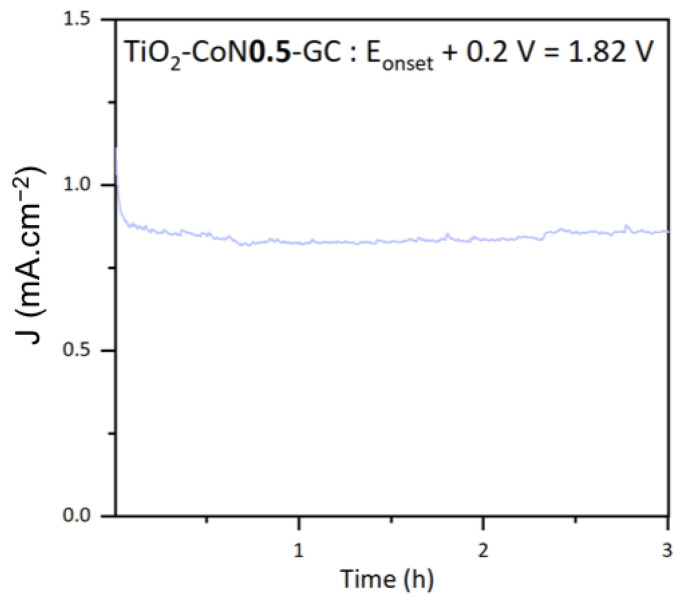
Photocurrent curve for the TiO_2_-CoN0.5 thin film.

The stability of the thin films was examined using photocurrent measurements. The stability of the top-performing TiO_2_-CoN0.5 sample is shown in Figure 11. The chosen potential was the onset potential +0.2 V, to ensure that non-negligible currents were obtained. At this potential, the thin film current density was stable at about 0.85 mA.cm^−2^ under xenon irradiation for 3 h. From this curve, the theoretical amount of hydrogen produced, $n_{H2}$, can be determined by utilizing Faraday’s law (5).

In this equation, the electric charge Q is the electric intensity i(t), integrated as a function of time *t*, $n_{e-}$ represents the number of electrons involved in the H_2_ reduction reaction, and F refers to Faraday’s constant. A theoretical yield of 0.3 µmol.min^−1^.cm^−2^ was calculated for the TiO_2_-CoN0.5.

(5) $$n_{H2}=\frac{\int_{0}^{t}i\left(t\right)\;dt}{n_{e-}\times F}=\frac{Q}{n_{e-}\times F}$$

## 3. Experimental Section

### 3.1. Synthesis Procedure

The starting materials, titanium (IV) isopropoxyde (TTIP, 99.999% trace metal basis), cobalt nitrate hexahydrate (Co(NO_3_)_2_•6 H_2_O, ≥98%), and nitric acid (HNO_3_, ACS reagent 70%) were all purchased from Sigma Aldrich (St. Louis, MO, USA). The three different polymeric biosurfactants are the same as those used in our previous work [35]: GBACoco (lot PF345, Surfactgreen, Rennes, France—named GC), GBAC18:1 (lot PF339, Surfactgreen, France—named GB), and Lansperse BIO868 (Bio-Loop Technology, Lankem, Dukinfield, UK—named BIO). They are all bio-based and 100% renewable. The solutions were prepared using a sol–gel method. For the TiO_2_, 10 mL of TTIP was mixed with 16.85 mL of HNO_3_ (2 mol.L^−1^), and for the TiO_2_-Co_3_O_4_, the cobalt nitrate was further added in two different molar ratios (HNO_3_/TTIP/Co(NO_3_)_2_•6 H_2_O = 1/1/0.25 and 1/1/0.5). Once the solution was stabilized, 1 g of surfactant was added. Then, the samples were shaped into two different forms: as powders for structural and morphological characterization, and as thin films for the other characterizations. The details of the dip coating, drying, and calcination steps have been reported in a previous paper [32]. Samples with ratios of 1/1/0.25 and 1/1/0.5 are hereafter referred to as TiO_2_-CoN0.25 and TiO_2_-CoN0.5, respectively.

### 3.2. Materials Characterizations

#### 3.2.1. Structural Characterization

Powder X-ray diffraction (XRD) was performed using an X’pert Pro (Pan Analytical, Malvern, UK) diffractometer with Cu-Kα radiation (Kα1 1.5405980 Å, Kα2 1.5444260 Å, ratio Kα1/Kα2 1.05), operating at 40 kV and 20 mA. Diffraction patterns were collected from 20° to 60° with a step size of 0.008° and a scan rate of 0.013° per second. Rietveld refinement was performed using FullProf with the CIF file of the anatase phase (CollCode 92363–Weirich T.E–2000), rutile phase (CollCode 93097–Ballirano P–2001) and spinel phase (CollCode 290720–Meena P.L–2013). The BET (Brunauer, Emmet, and Teller) specific surface area and mean pore size of the powders were measured using a Micromeritics ASAP-2010 (Norcross, GA, USA).

#### 3.2.2. Morphological and Chemical Characterizations

XPS analysis was performed on an ESCALAB 250 (Thermo Electron, Waltham, MA, USA) using a monochromatic source, Al Kα, as the excitation source, and the photoelectron spectra were calibrated as the binding energy relative to the energy of C-C of C1s at 284.8 eV. The relative error of the XPS atomic percentages is 10%. The thickness and surface area of the thin films were observed on a Hitachi S4800 (Santa Clara, CA, USA) with an AztecOne energy dispersive spectrometer. The error of the estimated thickness measurement and EDX ratio is 10–20%.

#### 3.2.3. Optical and Electrical Characterizations

UV absorption and band gap of the thin films were investigated using the integrating sphere spectrophotometer Shimadzu UV 3600 (Kyoto, Japan). A nanoscale conductive analysis of the thin films was performed on a Bruker Multimode 8 Atomic Force Microscope (AFM) setup equipped with a PtSi tip (radius of curvature, Rc = 27 nm, and spring constant, k = 1.8 N.m^−1^) using the conductive AFM (C-AFM) mode. A contact force of 33 nN and a current sensitivity module of 100 nA.V^−1^ were applied. The current was probed over a 2 µm × 0.5 µm area at three different locations on each sample with good consistency. A 384 × 96 pixel matrix was used for imaging, giving a pixel size of 5.2 nm × 5.2 nm.

#### 3.2.4. Photoelectrochemical Characterizations

Cyclic voltammetry and photocurrent experiments were monitored using a Solartron SI 1287 (Shildon, UK) and the impedance spectroscopy using a Solartron SI 1260. The photoelectrochemical cell was supplied by Pine Research Co., Durham, NC, USA, and was equipped with a quartz window. The electrolyte was a solution of 0.01 mol.L^−1^ NaOH–0.1 mol.L^−1^ Na_2_SO_4_, the Ag/AgCl electrode was used as the reference electrode, and the glass carbon as the counter electrode. Cyclic voltammetry curves were measured from 0.0 V/RHE to 2.0 V/RHE at a scan rate of 50 mV.s^−1^. The stability of the thin films were investigated by applying the onset potential + 0.2 V for 3 h. The interfacial conductivity of the thin films were measured in the dark at the onset potential over a frequency range from 0.1 Hz to 106 Hz, using an AC voltage of 10 mV amplitude. A 75 W LOT Quantum Design (San Diego, CA, USA) xenon lamp with a wavelength range of 250–2700 nm and a power density of 3 mW.cm^−2^ was used for the illumination.

## 4. Conclusions

The synthesis of TiO_2_-Co_3_O_4_/Co(OH)_2_ hybrid materials using the sol–gel method has made it possible to highlight the effects of cobalt on the properties of TiO_2_ thin films. First, in terms of the structural and textural properties, an inversion of the dominance of the anatase and rutile phases in the TiO_2_ powders before and after the addition of cobalt oxide was observed: the rutile phase was dominant in the absence of cobalt, whereas it was the anatase phase in the presence of cobalt. A homogenization of the crystallite and pore size, and an increase in the specific surface area of the hybrid powders were also observed. Second, with the optical characterization of the thin films using UV-Vis-NIR spectroscopy, it was shown that an increase in the visible absorption of the thin films is possible with the addition of cobalt oxide, and increasingly so with an increasing Co/Ti ratio. Third, the electronic properties of the thin films were characterized in two different ways: using C-AFM (at the nanoscale for volume analysis) and using EIS (at the macroscale for surface analysis). These analyses confirmed the heterogeneity of the electrical properties of the hybrid thin films due to the incorporation of cobalt oxide. Co_3_O_4_ in bulk did not improve the electrical conductivity of the materials, while Co(OH)_2_ on the surface of the films induced a significant decrease in the charge transfer resistance. Finally, the presence of cobalt enhanced the photoelectrocatalytic properties of the composite thin films. The current density of the cobalt-free thin films increased by a factor of 30, resulting in a maximum current of 1.570 mA.cm^−2^ at 2 V for the TiO_2_-CoN0.5 thin film with the highest Ti/Co ratio. The thin film’s stability was demonstrated for 3 h under xenon illumination, enabling it to theoretically produce 0.3 µmol.min^−1^.cm^−2^ of H_2_.

Given the feasibility of this synthesis method, it should be possible to explore the use of other metal oxide types, such as BiVO_4_ or Fe_2_O_3_, in a sol–gel one-pot synthesis to investigate the advantages of such a heterojunction. Additionally, future work will focus on coupling real-time H_2_ production with the photodegradation of a model pollutant in an electrolyte to qualify these materials as photoanodes for a PEC cell operating with water from real effluents.

## Figures and Tables

**Table 1:** BET results.

Samples	BET Surface Area (m²/g)	Adsorption-Average Pore Width BET (nm)	Pore Size Distribution (nm)
TiO_2_-GC	101	2.66	Bimodal: 4 nm–11 nm
TiO₂-CoN0.25-GC	112	4.85	Bimodal: 3.5 nm–5 nm
TiO₂-CoN0.5-GC	126	4.27	Unimodal: 3.5 nm
TiO_2_-GB	31	4.95	Bimodal: 4 nm–60 nm
TiO₂-CoN0.25-GB	91	4.56	Bimodal: 3.5 nm–5 nm
TiO₂-CoN0.5-GB	97	4.86	Unimodal: 3.5 nm
TiO_2_-BIO	16	8.77	Bimodal: 3 nm–9 nm
TiO₂-CoN0.25-BIO	88	5.82	Bimodal: 3.5 nm–5 nm
TiO₂-CoN0.5-BIO	126	4.07	Unimodal: 3 nm

## Data Availability

Data are contained within the article and Supplementary Materials.

## Supplementary Materials

The following supporting information can be downloaded at: https://www.mdpi.com/article/10.3390/molecules28227599/s1, Figure S1: BET results with p/p0 being the ratio of the applied N_2_ pressure over the N_2_ relative vapor pressure: (i) isotherm of GC samples, (ii) isotherm of GB samples, and (iii) isotherm of BIO samples; Figure S2: Pore size distribution for the TiO_2_-CoN0.25 samples: (i) TiO_2_-CoN0.25-GC, (ii)TiO_2_-CoN0.25-GB, and (iii) TiO_2_-CoN0.25-BIO; Figure S3: Pore size distribution for the TiO_2_-CoN0.5 samples: (i) TiO_2_-CoN0.5-GC, (ii) TiO_2_-CoN0.5-GB, and (iii) TiO_2_-CoN0.5-BIO; Figure S4: Nyquist plot in the dark at the onset potential for (i) TiO_2_-GC, (ii) TiO_2_-CoN0.25-GC, and (iii) TiO_2_-CoN0.5-GC; Table S1: Rietveld refinement data; Table S2: Crystallite size using the Scherrer formula; Table S3: Results from C-AFM characterization; Table S4: Data from the electrochemical impedance spectroscopy.
