# Supplementary material for: Hybrid Photoelectrocatalytic TiO2-Co3O4/Co(OH)2 Materials Prepared from Bio-Based Surfactants for Water Splitting
Source: Molecules. 2023 Nov 15;28(22):7599. doi: 10.3390/molecules28227599 (PMC10673594; doi:10.3390/molecules28227599)
Supplement: Supplementary file 1 [file molecules-28-07599-s001.zip › molecules-2680265-supplementary.pdf]

Supporting Information

Hybrid photoelectrocatalytic TiO_2-Co_3O_4/$Co(OH)_2$ materials prepared from bio-based surfactants for water splitting

Fanny Duquet[1,*], Valérie Flaud[2], Christina Villeneuve-Faure[3], Matthieu Rivallin[1,*], Florence Rouessac[1] and Stéphanie Roualdès[1]

Table S1. Rietveld refinement data.

Samples	Anatase Phase (%)	Bragg R-factor anatase	Rutile phase (%)	Bragg R-factor rutile	Spinelle phase (%)	Bragg R-factor spinelle	Rwp
TiO_2-GC	22.4	3.70	77.6	2.74	-	-	6.50
TiO_2-GB	10.3	3.75	89.7	2.75	-	-	6.01
TiO_2-BIO	38.5	2.04	61.5	2.92	-	-	5.98
TiO_2-CoN0.25-GC	47.2	1.63	46.7	2.19	6.1	5.90	1.76
TiO_2-CoN0.25-GB	40.8	1.98	50.0	2.40	9.2	5.83	1.87
TiO_2-CoN0.25-BIO	33.1	1.99	52.0	2.54	14.9	2.48	1.85
TiO_2-CoN0.5-GC	37.2	1.26	32.9	2.31	29.9	1.46	1.60
TiO_2-CoN0.5-GB	42.8	1.73	25.3	3.84	31.9	1.95	1.65
TiO_2-CoN0.5-BIO	41.7	1.55	23.0	2.71	35.3	1.89	1.69

Table S2. Crystallite size by Debye-Scherrer formula.

Samples	Anatase phase (nm)	Rutile phase (nm)	Spinelle phase (nm)
TiO_2-GC	16.1	14.2	-
TiO_2-CoN0.25-GC	3.4	5.5	10.9
TiO_2-CoN0.5-GC	3.6	5.6	5.5
TiO_2-GB	6.4	7.7	-
TiO_2-CoN0.25-GB	4.1	6.0	10.3
TiO_2-CoN0.5-GB	4.4	6.7	6.4
TiO_2-BIO	14.3	12.0	-
TiO_2-CoN0.25-BIO	4.2	6.8	7.2
TiO_2-CoN0.5-BIO	3.8	5.5	5.8

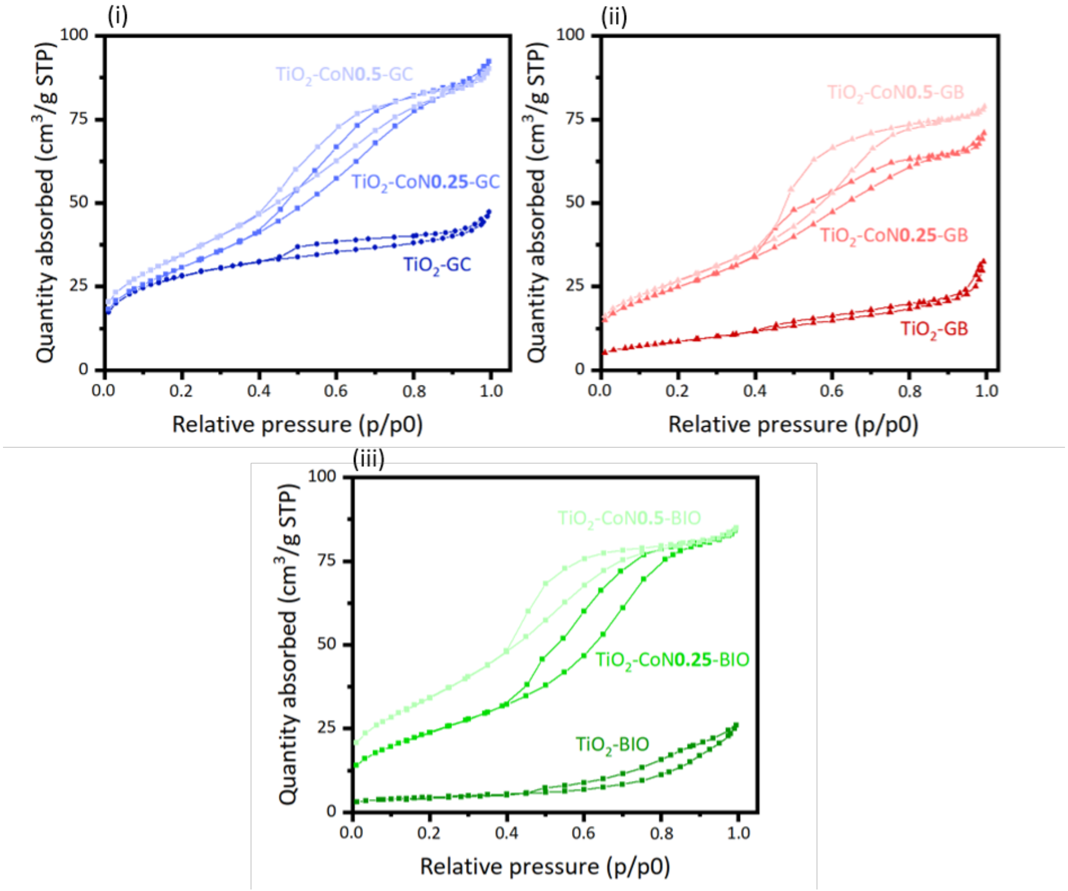

Figure S1. BET results with p/p0 being the ratio of the applied N_2 pressure over the N_2 relative vapor pressure. (i) Isotherm of GC samples, (ii) Isotherm of GB samples and (iii) Isotherm of BIO samples.

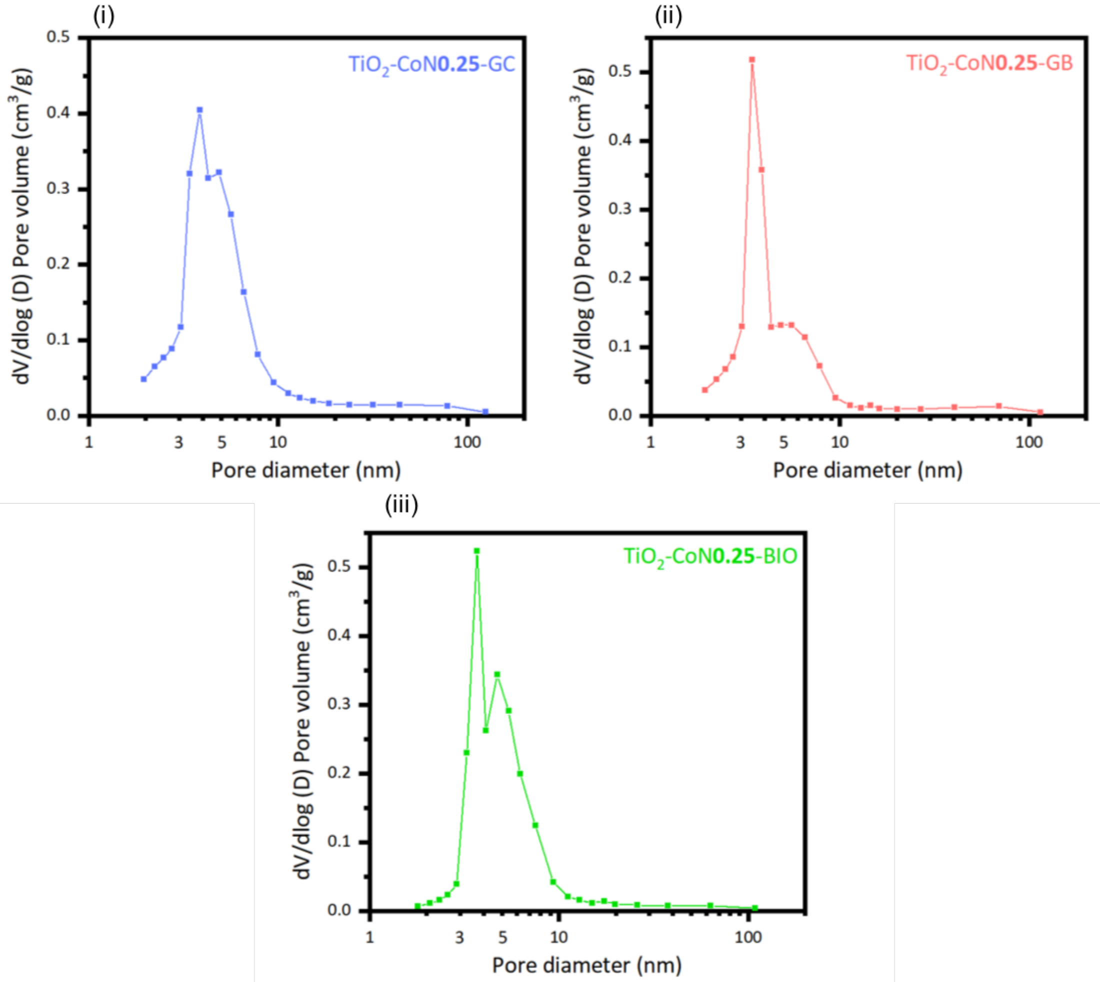

Figure S2. Pore size distribution for the TiO_2-CoN0.25 samples, (i) TiO_2-CoN0.25-GC, (ii)TiO_2-CoN0.25-GB and (iii) TiO_2-CoN0.25-BIO.

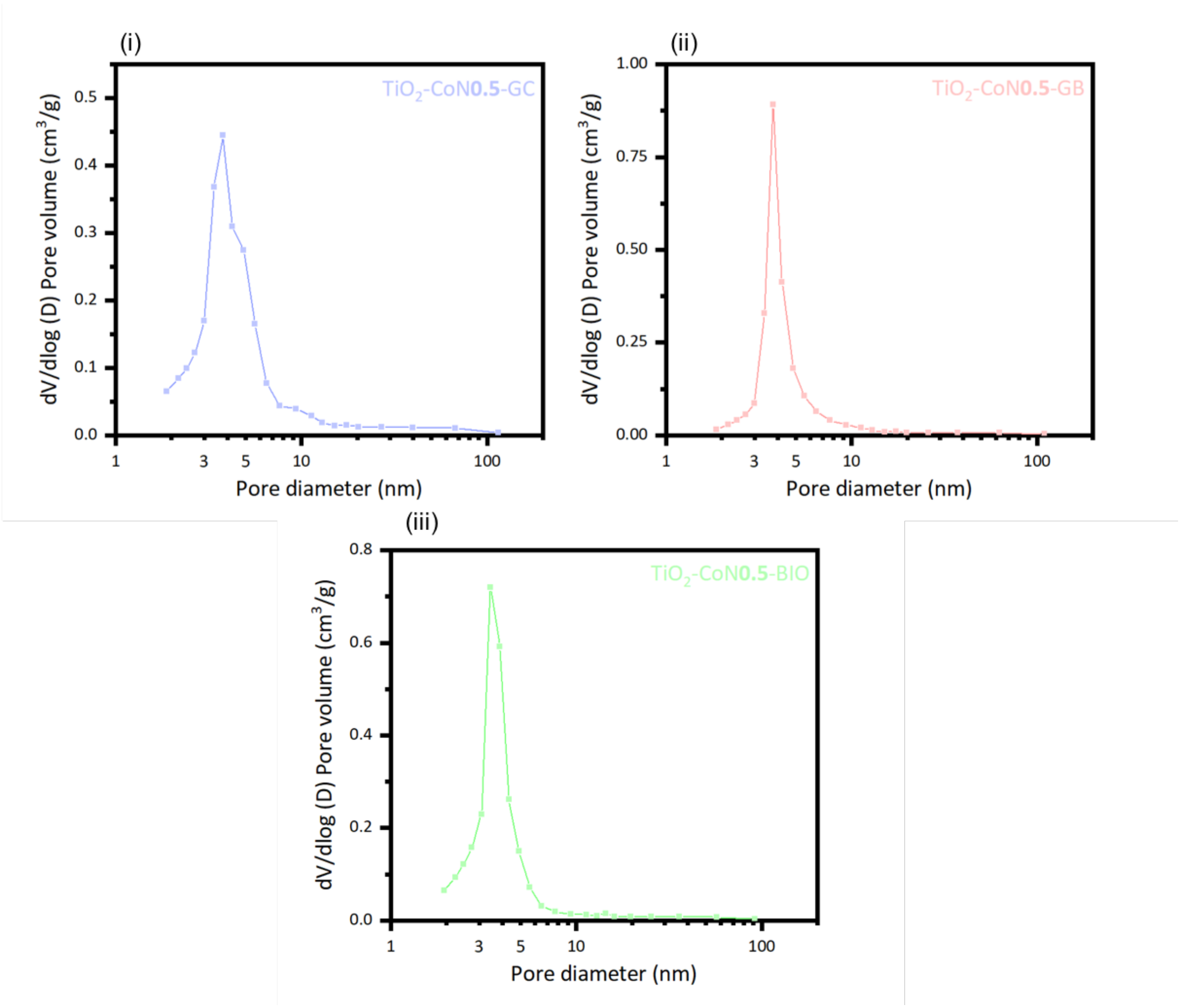

Figure S3. Pore size distribution for the TiO_2-CoN0.5 samples, (i) TiO_2-CoN0.5-GC, (ii) TiO_2-CoN0.5-GB and (iii) TiO_2-CoN0.5-BIO.

Table S3. Results from C-AFM characterisation.

Samples	Electrical field (10^6 V.m^{-1})	Associated Current (nA)	Conductive area (%)	Associated voltage (V)
TiO_2-GC	-9.52	-1.000	20	-5
TiO_2-CoN0.25-GC	-7.63	-0.035	85	-9
TiO_2-CoN0.5-GC	-7.59	-0.045 / -0.122	98	-9

Table S4. Data from the electrochemical impedance spectroscopy.

Samples	Rs (Ω)	Rp-1(Ω)	Onset potential (V)
TiO_2-GC	118	82	1.84
TiO_2-CoN0.25-GC	70	459	1.62
TiO_2-CoN0.5-GC	33	78	1.62

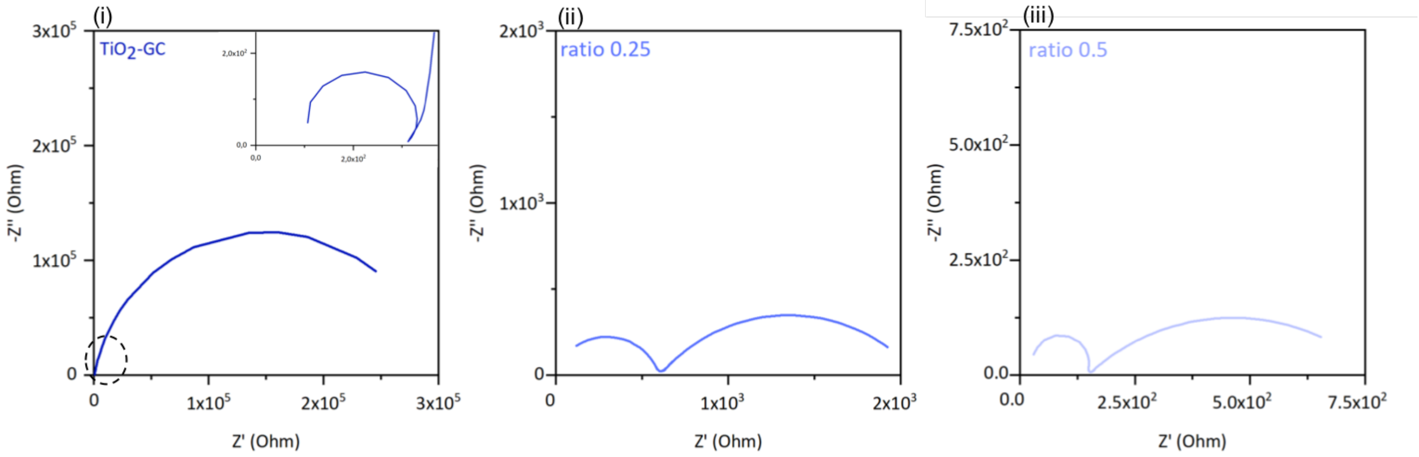

Figure S4. Nyquist plot in the dark at the onset potential for (i) TiO_2-GC (ii) TiO_2-CoN0.25-GC and (iii) TiO_2-CoN0.5-GC.
